# Low-Frequency Seismic Noise Properties in the Japanese Islands

**DOI:** 10.3390/e23040474

**Published:** 2021-04-16

**Authors:** Alexey Lyubushin

**Affiliations:** Institute of Physics of the Earth, Russian Academy of Sciences, 123242 Moscow, Russia; lyubushin@yandex.ru

**Keywords:** seismic noise, multifractals, entropy, principal component analysis, coherence, length of day, vector autoregression

## Abstract

The records of seismic noise in Japan for the period of 1997–2020, which includes the Tohoku seismic catastrophe on 11 March 2011, are considered. The following properties of noise are analyzed: The wavelet-based Donoho–Johnston index, the singularity spectrum support width, and the entropy of the wavelet coefficients. The question of whether precursors of strong earthquakes can be formulated on their basis is investigated. Attention is paid to the time interval after the Tohoku mega-earthquake to the trends in the mean properties of low-frequency seismic noise, which reflect the constant simplification of the statistical structure of seismic vibrations. Estimates of two-dimensional probability densities of extreme values are presented, which highlight the places in which extreme values of seismic noise properties are most often realized. The estimates of the probability densities of extreme values coincide with each other and have a maximum in the region: 30° N ≤ Lat ≤ 34° N, 136° E ≤ Lon≤ 140° E. The main conclusions of the conducted studies are that the preparation of a strong earthquake is accompanied by a simplification of the structure of seismic noise. It is shown that bursts of coherence between the time series of the day length and the noise properties within annual time window precede bursts of released seismic energy. The value of the lag in the release of seismic energy relative to bursts of coherence is about 1.5 years, which can be used to declare a time interval of high seismic hazard after reaching the peak of coherence.

## 1. Introduction

The article is devoted to the processing of seismic noise data recorded at the network F-net of stations on the Japanese Islands for 24 years, 1997–2020. During this time period, on 11 March 2011, a mega-earthquake with a magnitude of 9.1 occurred in Japan. Japan is a region with a dense, open-access seismic network F-net provided by the National Research Institute for Earth Science and Disaster Resilience (NIED). This makes it possible to test various hypotheses about how the preparation of a strong seismic event can affect the statistical properties of seismic noise.

In works [1,2,3,4,5,6], it was shown that the processes of earthquake preparation are preceded by certain changes in the statistical structure of seismic noise. The main changes are in the simplification of noise, namely in the growth of entropy and loss of multifractality. The main sources of seismic noise energy are not earthquakes, but the movements of cyclones in the atmosphere and the impact of ocean waves on the shelf and coast [7,8,9]. Thus, the sources of noise energy are located outside the earth’s crust. However, the crust is a medium for the propagation of seismic waves. As a result, processes inside the earth’s crust, including the preparation of strong seismic events, are reflected in changes in the properties of seismic noise.

The article investigates three properties of seismic noise: Entropy, determined through the distribution of the squares of orthogonal wavelet coefficients, the multifractal singularity spectrum support width, and the Donoho–Johnstone (DJ) index, defined as the proportion of the total number of wavelet coefficients that can be considered as "information carriers." These properties are estimated daily from seismic noise records at a network of stations. The choice of these parameters is due to the fact that their changes reflect the complication or simplification of the noise structure. With the simplification of the structure, the entropy increases, while the support width and the DJ index decrease. The methods outlined in the article are designed to detect spatial areas and time intervals, where and when the simplification of the noise structure is observed. These areas and time intervals are interpreted as manifestations of increased seismic hazard.

In addition, a possible trigger of the unevenness of the Earth’s rotation in relation to the release of seismic energy is being investigated. For this, the quadratic coherence between the length of the day and the first principal component of the analyzed properties of seismic noise is estimated within the annual sliding time window. The correlation function between bursts of coherence and the release of seismic energy turned out to be significantly shifted by time delays corresponding to the advance of the coherence maxima to strong earthquakes.

## 2. Description of Seismic Noise Properties

Minimum normalized entropy of wavelet coefficients En. Let x(t) be a random signal, and t=1,…,N is the discrete time index. Let $c_{j}^{\left(k\right)}$ be the wavelet coefficients of the analyzed signal. The superscript k is the number of the detail level of the orthogonal wavelet decomposition and the subscript j numbers the sequence of centers of time intervals in the vicinity of which the convolution of the signal with finite elements of the basis is calculated. The bases of 17 orthogonal Daubechies wavelets were used: 10 ordinary bases with a minimum support with the number of vanished moments from 1 to 10 and 7 Daubechies symlets [10] with the number of vanishing moments from 4 to 10. For each of the bases, the normalized entropy of the distribution of the squares of the coefficients was calculated.

(1) $$En=-\sum_{k=1}^{m}\sum_{j=1}^{M_{k}}p_{j}^{\left(k\right)}\ln p_{j}^{\left(k\right)}/\ln N_{r}\;,\;p_{j}^{\left(k\right)}=\;{\left|c_{j}^{\left(k\right)}\right|}^{2}/\sum_{l,i}{\left|c_{i}^{\left(l\right)}\right|}^{2}$$

where m is the number of levels of detail accepted for consideration; $M_{k}$ is the number of wavelet coefficients at the detail with the number k. The number of levels m depends on the length N of the analyzed sample. For example, if $N=2^{m}$, then m=n, $M_{k}=2^{\left(n-k\right)}$. The condition $N=2^{m}$ is necessary to apply the fast wavelet transform. If the length of N is not equal to a power of two, then the signal x(t) is padded with zeros to the minimum length L, which is greater than or equal to N: $L=2^{m}\geq N$. In this case, among the number $M_{k}=2^{\left(n-k\right)}$ of all wavelet coefficients at the level k, only $N\cdot 2^{-k}$ coefficients correspond to the decomposition of the real signal, while the remaining coefficients are equal to zero due to the addition of zeros to the signal x(t). Thus, in formula (1) $M_{k}=N\cdot 2^{-k}$ and only “real” coefficients $c_{j}^{\left(k\right)}$ are used to calculate the entropy. The number $N_{r}$ in formula (1) is equal to the number of “real” coefficients, that is, $N_{r}=\sum_{k=1}^{\;m}M_{k}$. The optimal wavelet basis is found as those that provide minimum to entropy among all 17 tested bases: En→min. By construction, 0≤En≤1.

The minimum entropy (1) was proposed in [11]. In works [3,5,6,11,12,13], it was used to analyze the predictive properties of seismic noise. Entropy (1) has common features with the multiscale entropy considered in [14,15]. The multiscale follows from the use of the wavelet transform, which provides decomposition into discrete energetic atoms ${\left|c_{j}^{\left(k\right)}\right|}^{2}$. A similar entropy construction was proposed in [16,17,18] for the natural time method. In [19,20], the non-extensive Tsallis entropy was used to study seismic noise.

Donoho–Johnstone index γ. In wavelet filtering, there is a procedure of “thresholding”, which means setting wavelet coefficients that are smallest by the absolute values to zero [10,21]. An assumption that noise is mainly accumulated at the first detail level is accepted. The variance of the wavelet coefficients is equal to the variance of the initial signal following from orthogonality of the wavelet transform. Thus, the noise standard deviation σ could be calculated as standard deviation of the wavelet coefficients at the first detail level. In order to provide a robust estimate, the median of absolute wavelet coefficients at the first detail level is used

(2) $$\sigma =med\{\;|c_{k}^{\left(1\right)}|\;,\;k=1,\ldots ,N/2\}/0.6745$$

where N/2 is the number of these coefficients. The formula (2) follows from the relation between median and standard deviation for normal random value: Med≈0.6745⋅σ. The estimate of standard deviation σ from formula (2) determines the quantity $\sigma \sqrt{2\cdot \ln N}$ as a “natural” threshold for separating the noisy wavelet coefficients [10,21]. The formula $\sigma \sqrt{2\cdot \ln N}$ is following from asymptotic probability of maximal deviations of Gaussian white noise [10]. Let us define the index γ, 0<γ<1 as the ratio of number of wavelet coefficients satisfying inequality $|c_{k}|\;>\;\sigma \sqrt{2\cdot \ln N}$ to the total number N of wavelet coefficients. The index γ is calculated for the optimal wavelet basis, which was found from minimum of entropy (1). The lower the index γ, the noisier the signal.

Singularity spectrum support width Δα. The measure of variability $\mu_{X}(t,\delta )$ of the random signal x(t) on the time interval [t,t+δ] is defined as its range: $\mu_{x}(t,\delta )=\underset{t\leq u\leq t+\delta }{\max}x(u)-\underset{t\leq u\leq t+\delta }{\min}x(u)$. Let us consider the mean value of its power degree q: $M(\delta ,q)=M[{\left(\mu_{x}(t,\delta )\right)}^{q}]$. The signal is scale-invariant [22] if $M(\delta ,q)\sim \;\delta^{\;\rho (q)}$ when δ→0, that is, the following limit exists:

(3) $$\rho (q)=\underset{\delta \to 0}{\lim}\left(\ln M(\delta ,q)/\ln\delta \right)$$

The process is monofractal if ρ(q)=Hq, where H=const, 0<H<1; otherwise, when ρ(q) is a nonlinear concave function of q, the signal is multifractal. The value of ρ(q) for a finite sample x(t), t=0,1,…,N−1 could be calculated using the method of detrended fluctuation analysis (DFA) [23,24], which was modified for estimating multifractals in [25]. The time series is split into adjacent intervals of length s:

(4) $$I_{k}^{\left(s\right)}=\{t:\;1+(k-1)s\leq t\leq ks,\;k=1,\ldots ,[N/s]\}$$

Let us consider a part of signal x(t), corresponding to interval $I_{k}^{\left(s\right)}$:

(5) $$y_{k}^{\left(s\right)}(t)=x((k-1)s+t),\;t=1,\ldots ,s$$

Let us fit a polynomial of the order m $p_{\;k}^{\left(s,\;m\right)}(t)$ to the signal $y_{k}^{\left(s\right)}(t)$ and consider the deflections:

(6) $$\Delta y_{\;k}^{\left(s,m\right)}(t)=y_{k}^{\left(s\right)}(t)-p_{\;k}^{\left(s,\;m\right)}(t),\;t=1,\ldots ,s$$

and the sum:

(7) $$Z^{\left(m\right)}(q,s)={\left(\sum_{k=1}^{\left[N/s\right]}{\left(\underset{1\leq t\leq s}{\max}\;\Delta y_{\;k}^{\left(s,m\right)}(t)-\underset{1\leq t\leq s}{\min}\Delta y_{\;k}^{\left(s,m\right)}(t)\right)}^{q}/\;[N/s]\right)}^{1/q}$$

The quantity (7) could be regarded as the estimate of ${\left(M(\delta_{s},q)\right)}^{1/q}$. Let us introduce the function h(q) as a linear regression coefficient between $\ln(Z^{\left(m\right)}(q,s))$ and ln(s): $Z^{\left(m\right)}(q,s)∼s^{h(q)}$ within scales ranging $s_{\min}\leq s\leq s_{\max}$. The minimum value of scale $s_{\min}$ within Formulae (4)–(7) was chosen to be 20 samples, and the maximum scale equals $s_{\max}=N/5$. For the monofractal signal h(q)=H=const, but in the general case, ρ(q)=qh(q). The multifractal singularity spectrum F(α) is defined as the fractal dimensionality of the set of time moments t for which the Hölder–Lipschitz exponent is equal to α, which means $|x(t+\delta )-x(t)|\;∼\;|\delta {|}^{\;\alpha },\;\delta \to 0$ [26]. Let us calculate a Gibbs sum:

(8) $$W(q,s)=\sum_{k=1}^{\left[N/s\right]}{\left(\underset{1\leq t\leq s}{\max}\;\Delta y_{\;k}^{\left(s,m\right)}(t)-\underset{1\leq t\leq s}{\min}\Delta y_{\;k}^{\left(s,m\right)}(t)\right)}^{q}$$

The mass exponent τ(q) is defined by the condition $W(q,s)∼s^{\tau (q)}$. A formula τ(q)=ρ(q)−1=qh(q)−1 follows from (7). The values of exponent q in the Formula (7) were taken from interval [−Q,+Q] where Q is some large number. The value Q=10 is used. The values $F(\alpha )=\underset{q\in [-Q,+Q]}{\min}(\alpha q-\tau (q))$ are calculated for $\alpha \in [A_{\min},A_{\max}]$ where $A_{\min}=\underset{q\in [-Q,+Q]}{\min}d\tau (q)/dq$ and $A_{\max}=\underset{q\in [-Q,+Q]}{\max}d\tau (q)/dq$. The derivative dτ(q)/dq is calculated numerically. The accuracy of its calculation is not very important, because this derivative is used for a rough determination of an a priori interval of α values. The value of $\alpha_{\min}$ and $\alpha_{\max}$ are determined as minimum and maximum values of α for which F(α)≥0. Thus, the spectrum F(α) is defined according to the formula:

(9) $$F(\alpha )=\max\;\{\;\underset{q\in [-Q,+Q]}{\min}(\alpha q-\tau (q)),\;0\;\}$$

Let us consider the estimates of singularity spectrum F(α) in a sliding window. For this case, its evolution can provide important information about the structure of the chaotic pulsations of the series. The support width of the singularity spectrum $\Delta \alpha =\alpha_{\max}-\alpha_{\min}$ is an important characteristic of the signal and it is regarded as a measure of variety of stochastic behavior.

Multifractal analysis is often used in geophysics [27,28]. The natural time approach [17] has its own toolboxes using multifractals and multiscale entropy for the analysis of seismicity [18,29,30,31]. The multifractal property Δα of seismic noise was used for the purposes of predicting earthquakes and assessing seismic hazard in [1,2,3,4,5,13,32]. The singularity spectrum support width Δα is used to study the behavior of various nonlinear systems. A decrease in the parameter Δα is a well-known effect that anticipates changes in the properties of biological and medical systems [33,34,35]. It was shown in [36] that the “loss of multifractality” is also universal in nature in physical systems.

## 3. Seismic Noise Data

**Figure 1:**
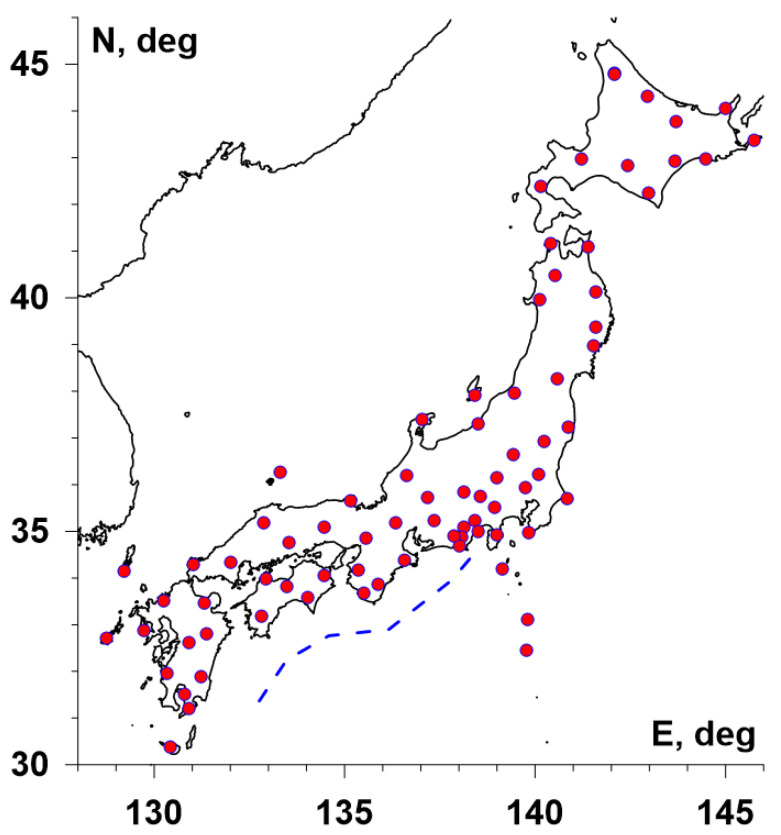
Positions of 78 seismic stations in Japan. The blue dashed line shows the position of the Nankai Deep Trench.

Vertical seismic oscillations data with sampling rate 1 Hz were used for the analysis. These data are accessible from the source [37] for 78 seismic stations of the network F-net in Japan. For the analysis, a time interval from the beginning of 1997 up to 31 March 2021 was selected. Figure 1 presents positions of the network stations and the location of the Nankai Deep Trench, which is the northern boundary of the Philippine tectonic plate.

The seismic data with a sampling frequency of 1 Hz were reduced to a time step of 1 minute by calculating the mean values in adjacent time intervals of 60 values. The seismic records from each station after coming to a 1-minute sampling time step were split into time fragments of the length of 1 day (1440 samples) and for each fragment, parameters (En,γ,Δα) of daily seismic noise waveforms were calculated. Scale-dependent trends in the Formulae (6) and (7) were removed by polynomials of the eighth order. Removing trends from seismic noise waveforms by the polynomial of the eighth order was used before computing entropy En and index γ in each daily time window. Thus, the time series of (En,γ,Δα) values with a sampling time step of 1 day was obtained from each of the seismic stations, which are presented in Figure 1.

**Figure 2:**
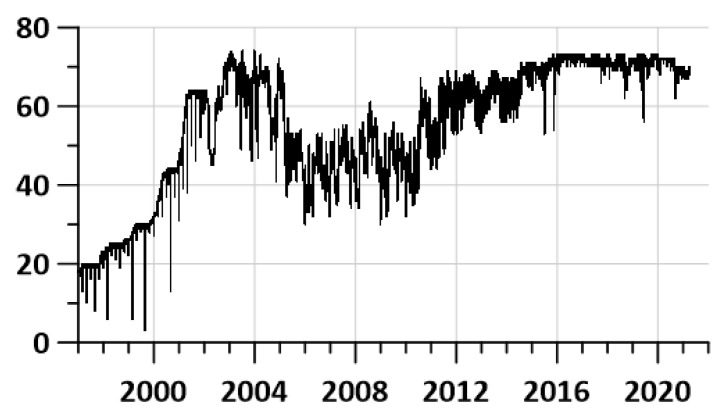
Daily number of working stations.

Figure 2 presents the graph of working stations each day during the considered time interval. The seismic station is considered working within the current day if there are no gaps during this day. One can see that the number of operable stations at the initial time fragment (1997–2001) is rather small. This influences the reliability of further estimates. Nevertheless, the result of data processing should not be removed from the analysis. Particularly, median values of seismic noise properties are rather stable to the number of working stations.

The initial seismic records with a 1 Hz sampling rate were taken “as is”, which includes background seismic oscillations and records immediately after earthquakes. It is important to underline that these 1 Hz records were transformed into a sampling time step of 1 min by averaging within adjacent time fragments of the length of 60 values. This smoothing operation gets rid of the influence of high-frequency immediate reactions of earthquakes.

The issue of predicting of strong earthquakes in Japan using entropy and multifractal properties of seismic noise was investigated in [1,2,3,4,5]. The natural time approach was applied to estimate seismic danger in Japan in [38,39].

## 4. Averaged Maps of Seismic Noise Properties

Let us consider the regular grid of the size 30 × 30 nodes covering the region with latitudes between 28° N and 46° N and longitudes between 128° E and 146° E (see Figure 1). Let U be any value of Δα, En or γ. For each node (i,j) of the grid and for each day t, the five nearest operable seismic stations are found, which provides five values of U. Let us take a median $U_{ij}^{\left(t\right)}$ of these values. The values $U_{ij}^{\left(t\right)}$ form an “elementary” daily map. These daily maps could be averaged:

(10) $${\bar{U}}_{ij}(t_{0},t_{1})=\sum_{t=t_{0}}^{t_{1}}U_{ij}^{\left(t\right)}/(t_{1}-t_{0}+1)$$

for daily time indexes t between two given dates $t_{0}$ and $t_{1}$.

**Figure 3:**
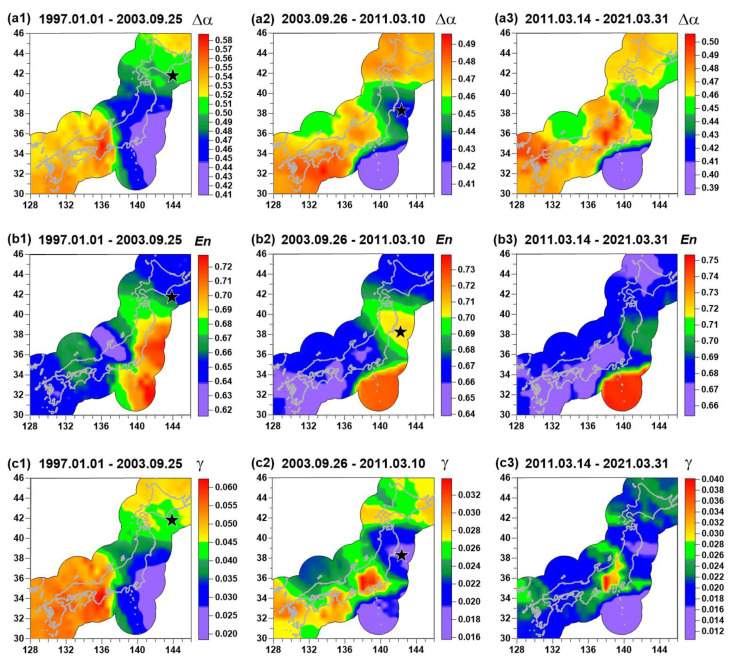
Averaged maps of seismic noise parameters Δα (**a1**–**a3**), En (**b1**–**b3**) and γ (**c1**–**c3**), calculated for three time intervals (1 January 1997–25 September 2003, 26 September 2003–10 October 2011, and 14 March 2011–31 March 2021). Black stars indicate hypocenters of two strong earthquakes: 25 September 2003, M = 8.3 and 11 March 2011, M = 9.1. The spatial distribution of seismic noise properties is shown only in the vicinity of the Japanese Islands in the union of circles with a radius of 250 km, built around each seismic station.

Figure 3 presents averaged maps of (En,γ,Δα) for adjacent time intervals: From the beginning of 1997 up to 25 of September 2003, the day of an earthquake with magnitude 8.3 near Hokkaido; from 26 of September 2003 up to 10 of March 2011, the day before the Tohoku mega-earthquake 11 of March with a magnitude 9.1l; and from 14 of March 2011 (3 days after seismic shock of 11 March of 2011) up to 31 of March 2021. The spatial distribution of seismic noise properties is shown only in the vicinity of the Japanese Islands in the union of circles with a radius of 250 km, built around each seismic station. During the considered time interval (1 January 1997–31 March 2021) only these two earthquakes with magnitude above 8 occurred at Japanese Islands. That is why these two events were taken as characteristic time markers.

The averaged map of Δα before the Tohoku earthquake is presented in Figure 3a2. One can notice that the region of future seismic events is extracted by relatively low values of Δα. If Figure 3a1,a2 are compared, one can see that after the earthquake on 25 September 2003, the domain with low values of singularity spectrum support width was split into two parts. The northern part turns out to be the region of the mega-earthquake on 11 March 2011 whereas the southern part preserves low values of Δα (Figure 3a2,a3).

Based on the assumption that low values of Δα correspond to high seismic danger, a hypothesis that the Tohoku earthquake drops only part of the accumulated tectonic energy from the southern region could be considered, and the region corresponding to the Nankai Trough could be regarded as the region of a future strong earthquake. Comparing the averaged maps of entropy En in Figure 3b1–b3 with similar maps of Δα in Figure 3a1–a3, one can notice that they are antipodes of each other. It means that high values of entropy En correspond to regions with high seismic danger. It should be noted that “high” and “low” values of seismic noise statistics are not considered absolute, but rather relative to the values within different time intervals. Each time interval is characterized by its own mean value, and differences with respect to mean value within the time interval define whether the value is “high” or “low” in the considered time interval.

A possible mechanism as to why low values of Δα and high values of En extract seismically dangerous regions was given in [2,3,4,5,6]. It is considered as the consequence of consolidation of small blocks of the Earth’s crust into the large one before the strong earthquake. Mutual movements of small blocks follow the existence of high-amplitude, irregular spikes in low-frequency seismic noise waveforms. After consolidation, these irregular spikes disappear, which cause the decrease of Δα and increase of entropy.

The peculiarities of spatial distribution of the wavelet-based index γ, as can be seen from a comparison of Figure 3a1–a3,c1–c3, basically coincides with the main features of spatial distribution of the Δα parameter. However, it should be noted that their calculation is based on completely different principles. Therefore, the γ parameter seems to provide additional information and their mutual consideration is important because estimations of these parameters are based on different approaches. It should be noticed that index γ in Figure 3c3 has low values in the region of the previous Tohoku events (compared with Figure 3c2. This could be the consequence of the preparation of two earthquakes that occur in the aftershocks region of the Tohoku event: Lat = 37.75° N, Lon = 141.71 ° E, M = 7.1 on 13 February 2021 and Lat = 38.47° N, Lon = 141.61 ° E, M = 7.0 on 20 March 2021. In addition, the Tohoku aftershocks region has lower values of Δα in Figure 3a3 and higher values of entropy in Figure 3b3 for the same reason.

## 5. Trends of Seismic Noise Properties

**Figure 4:**
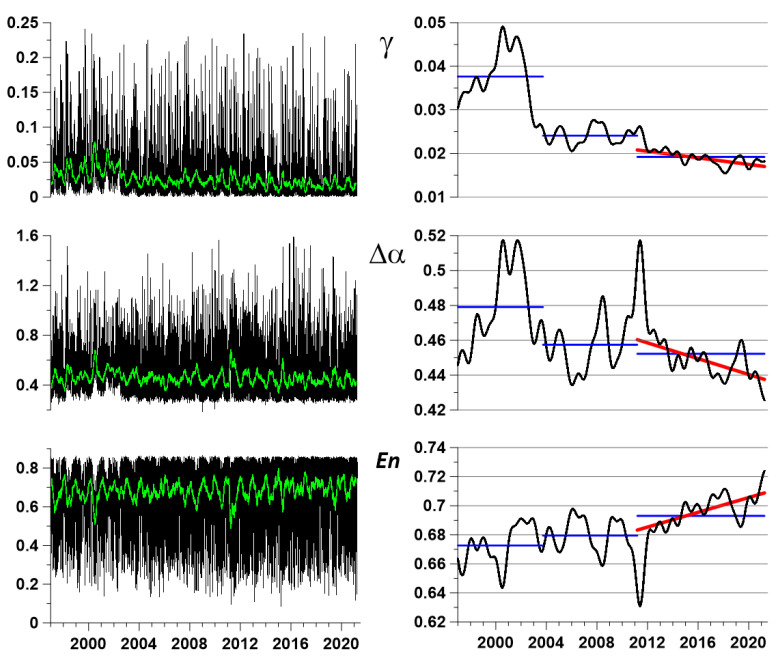
Left panel presents graphs of daily median values of seismic noise parameters γ, Δα and En, green lines are graphs of running average of the length 57 days. Right panel presents corresponding results of deep smoothing of median daily values by Gaussian kernel with bandwidth 182 days, red lines present linear trends of smoothed values after 11 March 2011. Horizontal blue lines present mean values of seismic noise statistics for three time intervals: 1 January 1997–25 September 2003, 26 September 2003–3 October 2011, and 14 March 2011–31 March 2021.

From the point of view of studying the processes of preparation of large earthquakes, the behavior of the integral characteristics of the field of seismic vibrations is of particular interest. As such characteristics, let us consider the median values of the properties of seismic noise, calculated daily for all operational stations of the seismic network. The three graphs on the left in Figure 4 show the median values of (En,γ,Δα), and green lines present running average values in a moving time window at the length of 57 days. The use of a moving average over a 57-day window is intended to facilitate visual perception of the daily median values of seismic noise properties. The window length of 57 days was chosen experimentally as a value that, on the one hand, smooths high-frequency pulsations of daily median seismic noise properties, and on the other hand, allows one to see the annual frequency of changes in these properties. The number 57 equals to double the length of the Moon month (28 days) plus 1. An additional unit is needed to make the window length odd.

To highlight possible predictive signs in the behavior of the median values of seismic noise properties, a deeper smoothing of high-amplitude random fluctuations should be performed. For this purpose, a kernel Gaussian smoothing [40,41] with a bandwidth of 182 days (half a year) will be applied. The result of this operation is shown in Figure 4 in the right column of the graphs. The red lines represent linear trends of smoothed values for the final observation interval, starting from 2012. From the behavior of the linear trends, it can be seen that, since 2012, there has been a systematic decrease in the singularity spectrum support width Δα and the DJ index γ, as well as an increase in the entropy En of seismic noise. It should be noted that the final Δα and γ values are less than any previous smoothed values, and the final entropy En value is superior to any previous value. Such behavior of the smoothed values is interpreted as an indicator of the growth of seismic hazard for the entire region of the Japanese Islands. The smoothed values of Δα and En at right panel of Figure 4 have a rather explicit reaction on the Tohoku mega-earthquake on 11 March 2011: The singularity spectrum width Δα increases whereas the entropy En decreases. As for the DJ index γ, its increasing is observed but its amplitude is rather weak. Thus, the statistic γ serves as some kind of additional characteristic of seismic noise to the “main” properties Δα and En. Nevertheless, the Pearson correlation coefficient between smoothed γ and Δα curves in the right panel of Figure 4 equals 0.77. Such a high value of correlation points out the fact that both these statistics reflect similar variations in seismic noise structure. Besides smoothed curves, it is interesting to compare simple mean values that are calculated for the sequence of three time intervals, which are discussed in Figure 3. These mean values are presented in the right panel of Figure 4 by blue horizontal lines. One can notice that mean values of γ and Δα progressively decrease, whereas the mean value of entropy En increases. This fact is interpreted as the general seismic danger in Japan permanently increasing.

## 6. Maps of Probability Densities for Extreme Values

Let us consider values of the parameter as a function of 2D vectors of longitudes and latitudes $z_{ij}=(x_{i},y_{j})$ of nodes (i,j) explicitly: $U_{ij}^{\left(t\right)}\equiv U^{\left(t\right)}(z_{ij})$. For each daily “elementary map” with a discrete time index t let us find coordinates $z_{\;mn}^{\left(t\right)}=(x_{m}^{\left(t\right)},y_{n}^{\left(t\right)})$ of the nodes where U attains a given number $n_{m}$ of extreme values with respect to all other nodes of the regular grid. If U=Δα or U=γ, then the minimum values will be sought. Otherwise, for U=En, the maximum values will be sought. Further on, $n_{m}=10$ extreme values are used. The cloud of 2D vectors $z_{\;mn}^{\left(t\right)}$, which are regarded within some time interval $t\in [t_{0},t_{1}]$, forms some random set. Let us estimate their 2D probability distribution function for each node $z_{ij}$ of the regular grid. For this purpose, the Parzen–Rosenblatt estimate with Gaussian kernel function [41] will be applied:

(11) $$p(z_{ij}|t_{\;0},t_{\;1})=\frac{1}{2\pi n_{m}h^{2}(t_{\;1}-t_{\;0}+1)}\sum_{t=t_{\;0}}^{t_{\;1}}\sum_{mn}\exp\left(-\frac{|z_{ij}-z_{\;mn}^{\left(t\right)}{|}^{2}}{2h^{2}}\right)$$

**Figure 5:**
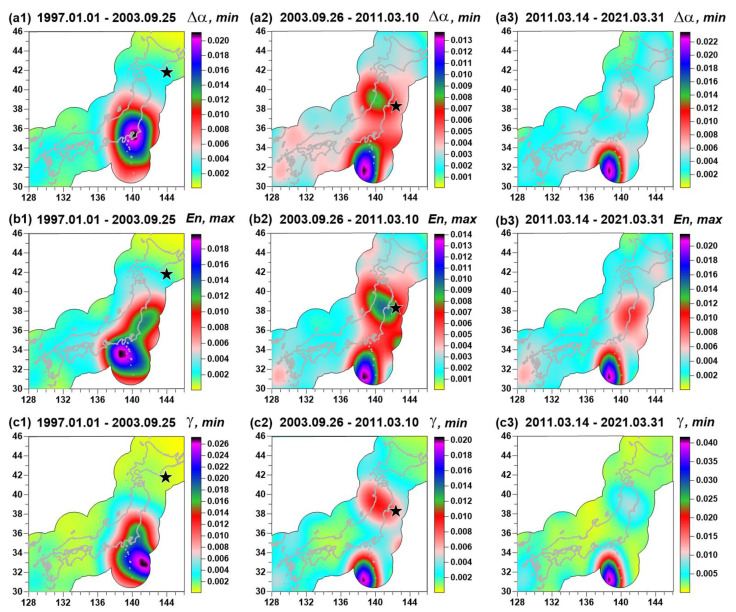
Maps of probability densities of extreme values, minimums for Δα, γ and maximums En, estimated for three time intervals (1 January 1997–25 September 2003, 26 September 2003–10 October 2011, and 14 March 2011–31 March 2021). Black stars indicate hypocenters of two strong earthquakes: 25 September 2003, M = 8.3 and 11 March 2011, M = 9.1. The distribution of probability densities of extreme values of seismic noise properties is shown only in the vicinity of the Japanese Islands in the union of circles with a radius of 250 km, built around each seismic station.

Here, h is the radius of kernel averaging (smoothing bandwidth), $t_{\;0},t_{\;1}$ are intege indices that numerate daily “elementary” maps. Thus, $\left(t_{\;1}-t_{\;0}+1\right)$ is the number of daily maps within the considered time interval. The smoothing bandwidth h = 1° was used. Figure 5 presents maps of probability density estimate (11) for time indices t corresponding to three time fragments similar to the maps presented at Figure 2. Kernel estimates (11) of the probability densities of extreme values of statistics of random fluctuations of geophysical fields in a moving time window were used in [6,42]. The distribution of probability densities of extreme values of seismic noise properties is shown only in the vicinity of the Japanese Islands in the union of circles with a radius of 250 km, built around each seismic station.

The main purpose of constructing two-dimensional maps of the probability distribution of extreme values of the studied statistics is an attempt to more accurately localize those areas where their minima or maxima are most often realized, compared to using simple maps of property values, similar to those shown in Figure 3. The probability density distribution maps of extreme values are presented in Figure 5 for the same three time intervals as in Figure 2. From a comparison of Figure 3 and Figure 5, it is noticeable that the maxima of the probability densities distinguish compact regions. Note that for the maps in Figure 5a2,b2,c2 for the time interval 26 September 2003–10 March 2011.near the epicenter of the future Tohoku mega-earthquake, there is a spot with an increased probability of minimum values for the Δα and γ parameters and maximum entropy En values. Thus, the maxima of the probability density of extreme values in Figure 5 much more accurately indicate the area of an impending strong earthquake than simple maps of the distribution of seismic noise statistics in Figure 3. In addition, attention is drawn to the area of increased probabilities, which is visible both for the time interval 26 September 2003–10 March 2011 and for 14 March 2011–31 March 2021 in the range 30° N ≤ Lat ≤ 34° N and 136° E ≤ Lon ≤ 140° E. It corresponds to a significantly broader range of lower Δα and γ values and higher entropy En values in Figure 3. This area is interpreted as a possible epicenter for the next mega-earthquake. This hypothesis will be discussed in more details in the Discussion section.

It is noteworthy that the maps of the probability densities of extreme values of the properties of seismic noise in the left column of the maps in Figure 5 do not distinguish the epicenter of the 2003 earthquake. As a “justification”, one can cite the consideration that the presented method for assessing “seismic hazard spots” is applicable only for mega-earthquakes such as the Tohoku event on 11 March 2011. The 2003 event off the coast of Hokkaido with a magnitude of 8.3 has an energy of almost 1.5 orders of magnitude less than the energy of the Tohoku event. Besides that, this could be the consequence of the small number of operable seismic stations during the initial time fragment of 1997–2001 (see Figure 2).

## 7. First Principal Component in Moving Time Window and Estimates of Coherence Spectrum

Furthermore, the method of principal components [43] for aggregating median daily time series of (En,Δα,γ) into one scaled time series will be applied. This will be necessary for investigating the connection of seismic noise properties with the irregularity of Earth’s rotation. A modification of the principal components method, which was proposed in [5], will be used.

Let us consider several time series of general dimensionality m $P(t)={\left(P_{1}(t),\ldots ,P_{m}(t)\right)}^{T}$, t=0,1,… In our case m=3. It is necessary to estimate first principal component of P(t) in a moving time window of the length L samples. For this purpose, we will consider samples with time indices t under the condition s−L+1≤t≤s where s is the most right-hand end of time window. Correlation matrix Φ(s) of the size m×m is calculated according to formulae

(12) $$\Phi (s)=\left(\varphi_{\;ab}^{\left(s\right)}\right),\;\;\varphi_{\;ab}^{\left(s\right)}=\sum_{t=s-L+1}^{s}q_{\;a}^{\left(s\right)}(t)q_{\;b}^{\left(s\right)}(t)/L,\;a,b=1,\ldots ,m)$$

(13) $$q_{\;a}^{\left(s\right)}(t)=(P_{a}(t)-{\bar{P}}_{\;a}^{\left(s\right)})/\sigma_{\;a}^{\left(s\right)},\;{\bar{P}}_{\;a}^{\left(s\right)}=\sum_{t=s-L+1}^{s}P_{a}(t)/L,\;\;{\left(\sigma_{\;a}^{\left(s\right)}\right)}^{2}=\sum_{t=s-L+1}^{s}(P_{a}(t)-{\bar{P}}_{\;a}^{\left(s\right)}{)}^{2}/(L-1),\;a=1,\ldots ,m$$

Let $\theta^{\left(s\right)}={\left(\theta_{\;1}^{\left(s\right)},\ldots ,\theta_{\;m}^{\left(s\right)}\right)}^{T}$ be the eigenvector of the matrix Φ(s) with the maximum eigenvalue. Let us calculate

(14) $$\psi^{\left(s\right)}(t)=\sum_{\alpha =1}^{m}\theta_{\;a}^{\left(s\right)}\cdot q_{\;a}^{\left(s\right)}(t)$$

and define the scalar time series ψ(t) of the adaptive first principal component in a moving window according to the formula:

(15) $$\psi (t)=\{\begin{array}{l} \psi^{\left(L-1\right)}(t),\;0\leq t\leq (L-1)\\ \psi^{\left(t\right)}(t),\;t\geq L\;\;\;\; \end{array})$$

Formulae (12)–(15) are applied independently within each time window. According to them, in the first time window, the time series ψ(t) consists of L values calculated according to (14)–(15). In all subsequent time windows, ψ(t) corresponds to the single most right-hand end. Thus, outside the first time window ψ(t) depends on past values of P(t) only.

For further analysis, it is necessary to estimate coherence spectra between two time series in a moving time window. A parametric model of vector autoregression that has a better frequency resolution than Fourier-based methods for estimating the spectra and cross-spectra [44] will be used. For a time series X(t) of dimensionality q, the AR-model is given by the formula:

(16) $$X(t)+\sum_{k=1}^{p}\;B_{\;k}\cdot \;X(t-k)=\varepsilon (t)\;$$

Here, t is the discrete time index, p is the order of autoregression, $B_{\;k}$ is the matrix of autoregression coefficients of the size q×q, and ε(t) is the residual signal covariance matrix $P=M\{\varepsilon (t)\varepsilon^{T}(t)\}$ of size q×q. The matrices $B_{\;k}$ and P are calculated by the Durbin–Levinson procedure [44]. The parametric estimate of the spectral matrix is defined by the formula:

(17) $$S_{XX}\left(\omega \right)=\Phi^{-1}\left(\omega \right)\cdot P\cdot \;\Phi^{-H}\left(\omega \right),\Phi \left(\omega \right)=E+\overset{p}{\underset{k=1}{\sum }}B_{k}e^{-i\omega k}$$

where E is the unit size q×q matrix. For dimension q=2, the quadratic coherence spectrum is calculated according to the formula:

(18) $$\beta^{2}\left(\omega \right)={\left|S_{12}\left(\omega \right)\right|}^{2}/\left(S_{11}\left(\omega \right)\cdot S_{22}\left(\omega \right)\right)$$

Here, $\;S_{11}\left(\omega \right)$ and $S_{22}\left(\omega \right)$ are the diagonal elements of matrix (17) whereas $S_{12}(\omega )$ is cross-spectrum. The coherence estimation was performed using a fifth-order vector autoregressive model with preliminary removal of linear trends and transition to increments.

## 8. Connection with Irregularity of Earth’s Rotation

The irregular rotation of the Earth traditionally is explained by the influence of processes in the atmosphere [45]. At the same time, some researchers pointed out the connection between the irregular rotation of the Earth and seismicity [46,47]. The possible trigger mechanism of the influence of variations in the Earth’s rotation on the seismic process was investigated in [48]. According to such an interpretation, a question arises about the impact of the processes in the atmosphere through the irregular rotation of the Earth on the seismic process. Estimates of the influence of the strong earthquake on the length of days are given in [49].

**Figure 6:**
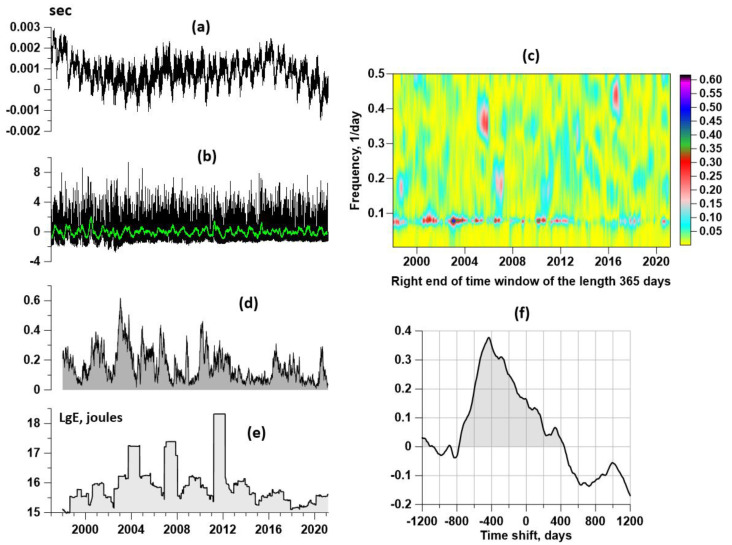
(**a**) Plot of length of day (LOD); (**b**) graph of first principal component of three seismic noise parameters calculated within the moving time window of 365 days; green line is a graph of running average of the length of 57 days; (**c**) time-frequency map of quadratic coherence between LOD and principal component (**b**) in a moving time window of the length of 365 days with a mutual shift of 3 days; (**d**) graph of the maximum values with respect to the frequencies of the squared coherence between LOD and the first principal component; (**e**) plot of the decimal logarithm of seismic energy released in the vicinity of the Japan Islands; (**f**) correlation function between the values of the logarithm of the released seismic energy and the maximums of coherence between the day length and the first principal component. Negative values of time shifts on graph (**f**) correspond to the lag in the release of seismic energy relative to bursts of coherence between LOD and seismic noise first principal component. Graphs (**d**) and (**e**) are plotted in dependence of the right end of time window of 365 days with an offset of 3 days.

Figure 6a presents a graph of the length of day (LOD), which was taken from the source [50]. Figure 6b is a graph of the first principal component of daily median values of seismic noise parameters (En,γ,Δα) (Figure 4, left panel), estimated in the time window of the length of 365 days. Figure 6c presents a time–frequency diagram of the evolution of squared coherence (18) between LOD and the first principal component in a moving time window the length of 365 days with an offset of 3 days. Bursts of coherence are concentrated within the narrow frequency band with periods from 11 up to 14 days. Figure 6d is a graph presenting maximum values with respect to the frequencies of the squared coherence. Figure 6e is a graph of the decimal logarithm of the seismic energy release (joules) in the vicinity of the Japan Islands: 28° N ≤ Lat ≤ 48° N, 128° E ≤ Lon ≤ 156° E. Information about seismicity was taken from the source [51].

In previous papers [11,52,53], maximum values of the coherence spectrum between LOD and daily median values of seismic noise properties were investigated in seeking the reasons for the existence of a break point in 2003 for trends and correlations of global seismic noise. It was shown that after 2003, trends of global seismic noise became typical for areas with increasing seismic hazard. Note that after the Sumatran mega-earthquake of 26 December 2004, M = 9, there was a sharp increase in the number of the strongest earthquakes around the world.

Thus, the structure of Figure 6 is the following. Figure 6c in the right-hand part of Figure 6 is a time-frequency map of the squared coherence function between two curves at the left-hand part presented by Figure 6a (LOD curve) and Figure 6b (first principal component of three daily median seismic noise properties). The map in Figure 6c illustrates a series of bursts of coherence within narrow frequency bands. This series of coherence bursts is presented as a one-dimensional graph in Figure 6d. The question of how much the bursts of coherence between LOD and the first principal component of the daily median properties of seismic noise can, on average, outpace the release of seismic energy is of interest. The estimate of this lag is presented in Figure 6e. To clarify this issue, the cross-correlation function for time shifts of ±1200 days was calculated. The graph of correlation function is presented in Figure 6f.

According to the correlation function estimate, one can notice that its values for negative time shifts significantly exceed the values for positive shifts. This confirms the fact that the release of seismic energy is delayed relative to bursts of the coherence measure. As for the lag time, it is estimated from the correlation maximum to be 432 days. This result also relates to the possibility of predicting strong earthquakes. Namely, if there is a burst of coherence between the LOD and the seismic noise properties, then this may be a harbinger of the strong release of seismic energy in about 1.5 years.

Although the value of correlation near 0.4 is rather small and it could not be an argument for a statistically significant linear connection between two random variables, I suppose that the main purpose of the correlation function estimate is establishing the fact that one variable shifted with respect to the other. The strong asymmetry of the correlation function in Figure 6f confirms the preceding of a coherence burst to a burst of seismic energy, which could be noticed visually by comparing the graphs in Figure 5d,e.

## 9. Discussion

The problem of a possible strong earthquake in the area of the Nankai Trough is traditional in Japanese seismology [54,55]. In [55], an estimate of the probability of 0.35–0.45 of the occurrence of an earthquake with a magnitude of more than 8.5 in this region during the time interval 2000–2010 is given. In [56], after the mega-earthquake of 11 March 2011, based on the analysis of GPS data, it was concluded that the probability of a strong earthquake to the south of the Tohoku aftershock region increased. Based on a retrospective analysis of seismic catalogs in [57], it was concluded that a magnitude 9 earthquake off the coast of Japan should not have come as a surprise. However, this event came as a surprise to traditional earthquake prediction methods. It led to an overestimation of the value of the maximum possible magnitude for seismic events in the Japan Trench, and in [58], an estimate is given up to a magnitude of 10.

A serious obstacle to the successful prediction of earthquakes is the presence of such a mechanism for the discharge of accumulated tectonic energy as “slow earthquakes” [59]. In fact, forecasting methods are only capable of identifying areas and time intervals where and when there is a noticeable accumulation of energy. The methods for studying low-frequency seismic noise discussed in the article can capture the accumulation of energy, which is associated with the consolidation of small blocks of the earth’s crust into large blocks and, as a consequence of this consolidation, the simplification of the statistical structure of the noise due to the disappearance of high-amplitude bursts arising from the mutual motion of small blocks. The stored energy can be discharged both as a result of an ordinary earthquake, and during a “slow earthquake”, the duration of which can be from several hours to several days. For the most part, slow earthquakes occur completely imperceptibly and are not recorded. Nevertheless, in terms of the efficiency of dissipation of accumulated tectonic energy, they are not inferior to ordinary “fast” earthquakes. Modern methods of analyzing geophysical data are still unable to give an estimate according to which of the two scenarios the accumulated energy will be released in. Due to this uncertainty in the practice of forecasting, there are many cases when the behavior of various characteristics of observations behaves similarly to previous cases before a seismic event; however, a new expected event does not occur, which means a possible release of energy according to a “quiet scenario”.

Identification of areas in which minima or maxima of seismic noise statistics are most often realized, like the maps in Figure 5, provides a dynamic assessment of seismic hazard. The regions of maxima of two-dimensional distribution densities of extreme values of seismic noise statistics can be called “seismic hazard spots”. Seismic hazard spots can arise and disappear without the manifestation in the form of an ordinary earthquake. At the same time, the detection of a stable ”seismic hazard patch” like an area 30° N ≤ Lat ≤ 34° N, 136° E ≤ Lon ≤ 140° E means that there are persistent tectonic causes, and such areas should be given close attention.

As for the estimation of the event time, this part of the earthquake forecast is the most difficult. At present, one can only talk about the assessment of the trend, that is, whether the seismic hazard is increasing or decreasing. In this regard, the linear trends presented in the right column of the graphs in Figure 3 indicate an increase in the seismic hazard of the next mega-earthquake. In addition, the graph of the cross-correlation function presented in Figure 6f can give an estimate of the time of the next large earthquake from the occurrence of a burst of the coherence function between the median properties of seismic noise and the time series of the length of day reflecting the irregular rotation of the Earth.

## 10. Conclusions

A method for analyzing continuous records of low-frequency seismic noise on a network of broadband stations in a seismically active region is presented. The method is based on the calculation of multifractal singularity spectrum support width and wavelet-based entropy and Donoho–Johnstone index in successive daily time intervals. Methods have been developed for assessing the spatial distribution of the values of these seismic noise properties and the identification of "seismic hazard spots" as areas of increased values of two-dimensional probability densities of extreme values of seismic noise statistics. The method is applied to the analysis of data from the F-net seismic network on the Japanese Islands for the time interval from the beginning of 1997 to the end of March 2021. The question of a possible trigger effect of the irregularity of Earth’s rotation on the seismic energy release is considered. An estimate of the lag between strong bursts of seismic energy release and bursts of coherence between the time series of the length of the day and the median values of seismic noise properties in Japan is obtained. The value of this lag is close to 1.5 years, which could be used for announcing a high seismic danger time interval after the peak of coherence is achieved.

## Data Availability

The open access data from the sources: http://www.fnet.bosai.go.jp/faq/?LANG=en, https://hpiers.obspm.fr/iers/eop/eopc04/eopc04.62-now, https://earthquake.usgs.gov/earthquakes/search/ were used. All data have access on 4 April 2021.
